# Prototype foamy virus elicits complete autophagy involving the ER stress-related UPR pathway

**DOI:** 10.1186/s12977-017-0341-x

**Published:** 2017-03-07

**Authors:** Peipei Yuan, Lanlan Dong, Qingqing Cheng, Shuang Wang, Zhi Li, Yan Sun, Song Han, Jun Yin, Biwen Peng, Xiaohua He, Wanhong Liu

**Affiliations:** 1 Hubei Province Key Laboratory of Allergy and Immunology, School of Basic Medical Sciences, Wuhan University, No. 185, Donghu Road, Wuchang District, Wuhan, 430071 China; 2 Hubei Provincial Key Laboratory of Developmentally Originated Disease, School of Basic Medical Sciences, Wuhan University, Wuhan, 430071 China; 3 Wuhan Ammunition Life Technology Co., Ltd, Wuhan, 430206 China; 4 College of Life Sciences, Shanxi Normal University, Xi’an, 710062 China

**Keywords:** PFV, Autophagy, ER stress, Viral replication

## Abstract

**Background:**

Prototype foamy virus (PFV) is a member of the Spumaretrovirinae subfamily of retroviruses, which maintains lifelong latent infection while being nonpathogenic to their natural hosts. Autophagy is a cell-programmed mechanism that plays a pivotal role in controlling homeostasis and defense against exotic pathogens. However, whether autophagy is the mechanism for host defense in PFV infection has not been investigated.

**Findings:**

Our results revealed that PFV infection induced the accumulation of autophagosomes and triggered complete autophagic flux in BHK-21 cells. PFV infection also altered endoplasmic reticulum (ER) homeostasis. The PERK, IRE1 and ATF6 pathways, all of which are components of the ER stress-related unfolded protein response (UPR), were activated in PFV-infected cells. In addition, accelerating autophagy suppressed PFV replication, and inhibition of autophagy promoted viral replication.

**Conclusions:**

Our data indicate that PFV infection can induce complete autophagy through activating the ER stress-related UPR pathway in BHK-21 cells. In turn, autophagy negatively regulates PFV replication.

**Electronic supplementary material:**

The online version of this article (doi:10.1186/s12977-017-0341-x) contains supplementary material, which is available to authorized users.

## Findings

Prototype foamy virus (PFV) is a member of the foamy viruses (FVs; also known as spumaviruses) belonging to a subfamily of the Retroviridae family [1]. In contrast to human immunodeficiency virus (HIV) and human T-cell leukemia virus (HTLV), FVs appear to be nonpathogenic in either naturally or accidentally infected hosts and maintain a lifelong infection in the host [2, 3]. Several host factors, such as Trim5α, APOBEC3G and Nmi have been studied as restrictors during PFV replication [4–8]. Previously, we also reported that Pirh2 inhibits PFV replication by degrading the viral trans-activator Tas via ubiquitination [9]. However, the mechanisms that PFV utilizes to maintain perpetual nonpathogenicity in host cells remain elusive.

Autophagy is a highly conserved catabolic pathway that maintains cellular homeostasis. As an intrinsic defense mechanism, host cells may utilize autophagy against invading viruses [10, 11]. Sagnier et al. reported that autophagy can be an antiviral process due to its degradation of the HIV-1 trans-activator Tat, which is a protein essential for viral replication [12]. Inhibition of autophagy could lead to increased viral replication and virulence for herpes simplex virus type-1 (HSV-1) and Sindbis viruses [13, 14]. In addition, autophagy is primarily antiviral for Japanese encephalitis virus (JEV) and might have implications for the disease progression and pathogenesis of JEV [15].

However, the relationship between PFV infection and autophagy remains unexplored. In this paper, we reported that PFV infection could induce autophagy and investigated the mechanism underlying this phenomenon.

### PFV infection triggers the accumulation of autophagosomes

**Fig. 1:**
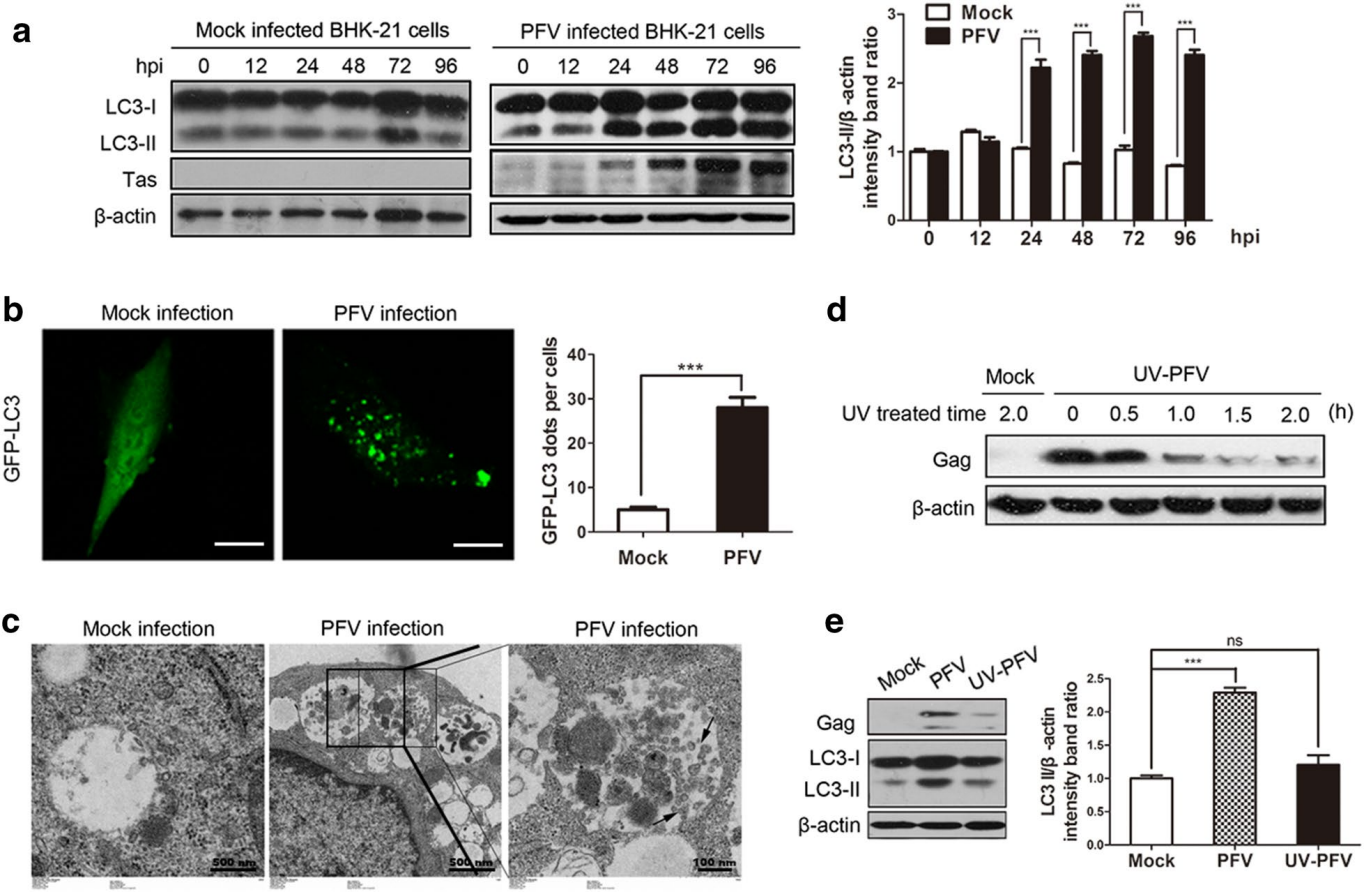
PFV infection promotes the accumulation of autophagosomes. **a** BHK-21 cells were infected with PFV to analyze LC3 protein expression by western blotting. After 1.5 h of virus absorption at 37 °C, the cells were further cultured in maintenance medium. The cells were infected with mock or PFV at an MOI of 0.5. After PFV infection, cell samples were harvested at 0, 12, 24, 48, 72 and 96 hpi, and cell extracts were blotted with anti-LC3 and anti-Tas antibodies. **b** GFP-LC3 dots were visualized via confocal microscopy. BHK-21 cells were transfected with GFP-LC3 plasmids for 24 h, followed by PFV infection (MOI = 0.5) or mock infection for 24 h, and the GFP-LC3 aggregates in the cells were assessed via confocal microscopy. *Scale bars* 10 μm. The *graph* shows the quantification of autophagosomes by calculating the average number of dots in 20 cells. **c** Autophagic vacuoles were detected via TEM. BHK-21 cells infected with PFV at an MOI of 0.5 were processed and analyzed at 24 hpi for the accumulation of autophagosomes via transmission electron microscopy. *Black frame* indicated representative autophagosomes and *arrow* indicated representative PFV capsid structures during PFV infection. *Scale bars* 500 and 100 nm. **d** BHK-21 cells were infected with UV-inactivated PFV, and the inactivated virus infectivity was confirmed by examining the viral structure protein Gag via western blot. Before infection, PFV were radiated by UV for 0, 0.5, 1.0, 1.5 and 2.0 h respectively. Mock-infected supernatant was also radiated with UV for 2.0 h before infecting cells. Then cells were infected with these UV-inactivated mock supernatant or PFV supernatant for 24 h. **e** BHK-21 cells were inoculated with normal PFV or UV-inactivated PFV (MOI = 0.5) for 24 h. Before infection, PFV and Mock supernatant were treated with UV for 1.5 h. Then, the cell samples were processed and blotted with anti-LC3 antibody. Quantitation of protein levels from the western blot by using Quantity one software (Bio-Rad); all data are representative of three independent experiments with triplicate samples. Significance was analyzed with a two-tailed Student’s *t* test. ^ns^ *P* > 0.05, ***P < 0.001

To determine whether PFV infection could induce autophagy, we used three experimental methods: (1) We examined LC3 conversion (from LC3-I to LC3-II), an important hallmark of autophagy, using western blotting analysis. (2) The punctate accumulation of LC3, another autophagy biomarker, which represents the recruitment of LC3-II to autophagic vacuoles was detected by confocal microscopy [16, 17]. (3) We also used transmission electron microscopy (TEM), an accepted gold standard method, to visually observe autophagosome formation in PFV-infected cells [16, 17]. First, baby hamster syrian kidney (BHK-21) cells were infected with PFV and collected at 0, 12, 24, 48, 72, 96 hours post-infection (hpi). The expression of the viral protein Tas was measured to track the progression of PFV infection. We found that the intensity of LC3-II was significantly increased in the cells infected with PFV at 24, 48, 72, 96 hpi compared to mock-infected cells (Fig. 1a), indicating that autophagy was indeed induced by PFV infection. Second, to further demonstrate that PFV infection could increase autophagosome formation, BHK-21 cells were transfected with the GFP-LC3 plasmid for 24 h and then infected with PFV. There was a significant increase in the percentage of cells with autophagosomes (GFP-LC3 dots) in PFV-infected cells relative to mock-infected cells (Fig. 1b). Third, PFV-infected cells also had observably increased double-membrane vesicles (Fig. 1c), which are morphologically typical characteristics of autophagic vacuoles. These results suggested that PFV infection could induce autophagy and autophagosome formation.

Next, we analyzed whether PFV replication is required for the induction of autophagy. PFV was inactivated by ultraviolet (UV) radiation, and its ability to induce autophagy was examined. As shown in Fig. 1d, synthesis of the PFV structural protein Gag was dramatically decreased in BHK-21 cells infected with UV-inactivated PFV which was treated with UV for 1.5 and 2.0 h. Meanwhile, the levels of both LC3-I and LC3-II were detected in BHK-21 cells infected with UV-inactivated PFV. LC3-II conversion was not increased in UV-PFV-infected BHK-21 cells similar to mock-treated cells. Conversely, there was an apparent conversion from LC3-I to LC3-II in BHK-21 cells infected with normal PFV (Fig. 1e). Although PFV infectivity could be inactivated by UV radiation, the Gag expression were not been eliminated completely as the Fig. 1c shown. It could be speculated that the UV-radiated PFV might not been full deactivated and might remain very low infectious. UV-inactivated PFV might cause host cell response, while there were no significant statistical differences in LC3-II conservation and LC3 accumulation in mock-infected and UV-PFV-infected groups (Additional file 1: Figure S1a, S1b, S1c). These results suggested that the replication of PFV was required for the induction of autophagy.

### PFV-induced autophagy is a complete autophagic process

**Fig. 2:**
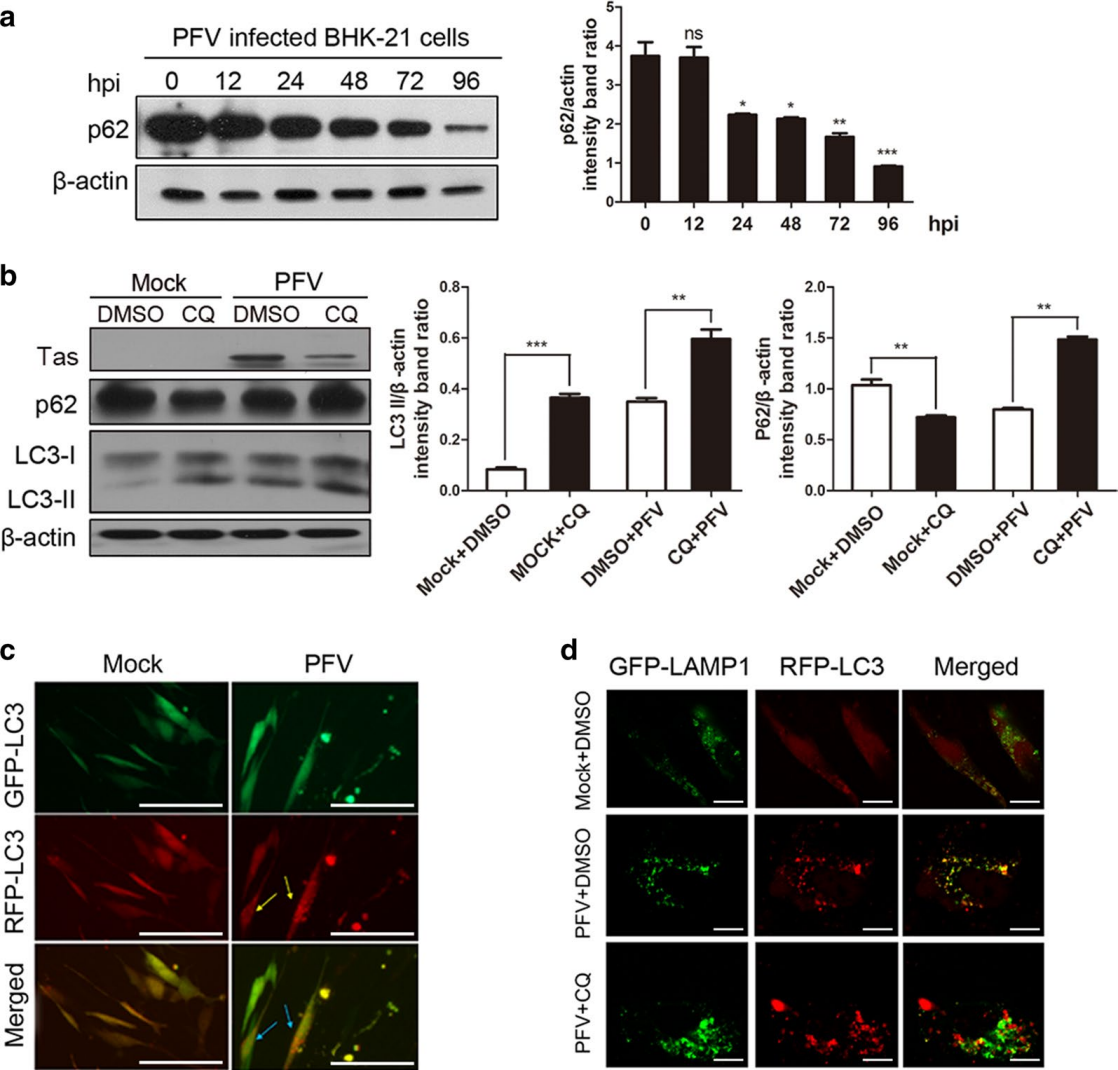
Measurement of autophagic flux in BHK-21 cells infected with PFV. **a** BHK-21 cells were infected with PFV to analyze p62 protein expression by western blotting. After 1.5 h of virus absorption at 37 °C, the cells were further cultured in maintenance medium. The cells were infected with PFV at an MOI of 0.5. After PFV infection, cell samples were harvested at 0, 12, 24, 48, 72 and 96 hpi, and cell extracts were blotted with anti-p62 antibody. **b** BHK-21 cells were pretreated with the autophagy inhibitor CQ (50 μM) for 4 h, followed by infection with mock or PFV at an MOI of 0.5. After 1.5 h of virus absorption at 37 °C, the cells were further cultured in maintenance medium in the absence or presence of CQ. At 24 h of infection with mock or PFV, the cells were subjected to western blotting using anti-LC3 and anti-p62 antibodies. **c** BHK-21 cells transfected with ptfLC3 were infected with mock or PFV (MOI = 0.5). The cells were collected, fixed, and visualized at 24 hpi using a confocal microscope. *Scale bars* 100 μm. **d** Co-localization of the GFP-LC3 and LAMP1 proteins in BHK-21 cells infected with PFV and treated with CQ or DMSO. The cells were cotransfected with GFP-LAMP1 and RFP-LC3 for 12 h and pretreated with CQ (50 μM) or DMSO control. Then cells were infected with mock or PFV at an MOI of 0.5 for 24 h. The protein localization was observed using a confocal microscope. *Scale bars* 10 μm. Significance was analyzed with a two-tailed Student’s *t* test. ^ns^ *P* > 0.05, *P < 0.05, **P < 0.01, ***P < 0.001

It is known that the accumulation of autophagosomes may be the result of either increased de novo autophagosome formation, which occurred as the autophagosomes fused with lysosomes in autophagic flux, (complete autophagy process) or disrupted trafficking to lysosomes for degradation (incomplete autophagy process) [11, 18, 19]. To illuminate whether the autophagy induced by PFV infection is a complete or incomplete autophagic process, we used three methods: (1) We measured the consumption of the p62 autophagy adaptor in PFV-infected BHK-21 cells. P62, whose degradation suggests a complete autophagic process, is frequently used to assess autophagic flux. (2) A plasmid containing an mRFP-GFP tandem fluorescent-tagged LC3 (ptfLC3) was used. This protein emitted GFP and mRFP signal before autophagosomes fusion with lysosomes and emitted only the mRFP signal during the complete autophagy process [20]. (3) We measured the co-localization of LC3 and lysosomal-associated membrane protein 1 (LAMP1). In the complete autophagic process, with the autophagosomes trafficking to lysosomes and forming autophagolysosomes, the yellow signal, which represents the co-localization of RFP-LC3 and GFP-LAMP1, can be observed by confocal fluorescence [21]. First, BHK-21 cells were infected with PFV and collected at 0, 12, 24, 48, 72, 96 hpi to detect p62 expression. In addition, we pretreated cells with chloroquine (CQ) to prevent fusion between autophagosomes and lysosomes in the late phase of autophagic flux before viral infection. As shown in Fig. 2a, the consumption of endogenous p62 could be observed with the progression of PFV infection relative to mock-infected or UV-inactivated PFV infected cells (Additional file 1: Figure S1a, S1b). Furthermore, the consumption of p62 could be rescued by CQ (Fig. 2b). And, when PFV-infected cells were treated with CQ, comparable LC3-II levels were observed to DMSO-treated control group. Second, a tandem probe mRFP-GFP tandem fluorescent-tagged LC3 plasmid, ptfLC3, was also used to detect autophagic flux. There were a large number of red autolysosome vacuoles in PFV-infected cells compared to the yellow signal in Mock-infected cells (Fig. 2c). Third, the GFP-LAMP1 and RFP-LC3 plasmids were cotransfected into BHK-21 cells to observe the distribution of autophagosomes and lysosomes in PFV-infected cells. The co-localization of LAMP1 and LC3 was evidently increased in PFV-infected cells compared to mock-infected cells (Fig. 2d). When cells were pretreated with CQ, which is an inhibitor that prevents autophagosome-lysosome fusion, PFV infection could not induce the co-localization of LAMP1 and LC3 (Fig. 2d). All together, these results supported the conclusion that PFV infection could trigger the complete autophagic process and induce the formation of autophagolysosomes by promoting autophagosomes maturation.

### ER stress contributes to PFV-induced autophagy

**Fig. 3:**
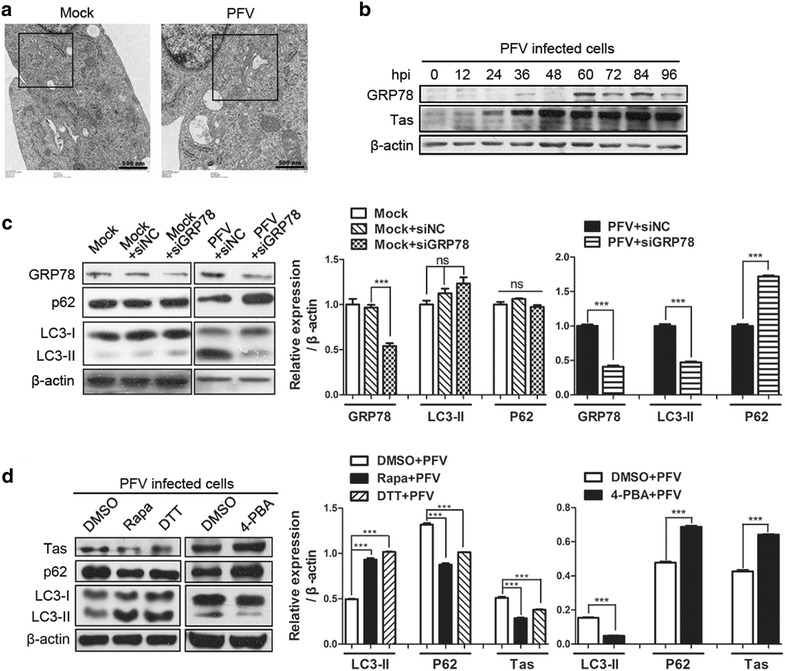
PFV triggers autophagy by activating ER stress. **a** Expansion of the ER was detected via transmission electron microscopy (TEM). BHK-21 cells infected with mock supernatant or PFV at an MOI of 0.5 were processed and analyzed at 24 hpi for the ER expansion via electron microscopy. *Black frames* indicated representative expanded ER lumen. *Scale bars* 500 nm. **b** BHK-21 cells were infected with PFV to analyze GRP78/Bip protein expression by western blotting. **c** BHK-21 cells were transfected with the siRNAs of GRP78 or siRNAs of Negative control for 36 h, followed by infection with mock supernatant or PFV at an MOI of 0.5 or mock infecting. After 1.5 h of virus absorption at 37 °C, the cells were further cultured in maintenance medium. At 24 h after infection with mock or PFV, the cells were subjected to western blotting using anti-GRP78, anti-P62 and anti-LC3 and antibodies. In *lane 1*, cells were only infected with mock supernatant and cultured in maintenance medium for 48 h. **d** Western blotting was used to analyze the expression of the viral protein Tas and LC3 in PFV-infected cells in the absence or presence of the ER stress inducer DTT (1 mM) or the ER stress inhibitor 4-PBA (1 mM). Rapa-treated (400 nM) cells were used as a positive control. Significance was analyzed with a two-tailed Student’s *t* test. ^ns^ *P* > 0.05, ***P < 0.001

The endoplasmic reticulum (ER) is an organelle found in eukaryotic cells for key functions, such as protein translation, folding and maturation. Moreover, the ER also acts as a sensor for cellular stress, such as the accumulation of abundant cellular proteins [22]. It was recently reported that autophagy is induced via the ER stress response [23–25]. On account of PFV-induced autophagy, which is related to its replication (Fig. 1), we wondered whether PFV could induce ER stress and subsequently regulate autophagy. We first used TEM to observe the ER lumen in PFV-infected cells. A greatly expanded ER lumen (a morphological marker of ER stress) could be observed in PFV-infected cells compared with mock-infected cells (Fig. 3a). Moreover, Glucose-regulated protein 78 (GRP78/Bip), a biomarker of ER stress, was significantly increased in PFV-infected BHK-21 cells relative to mock-infected or UV-inactivated PFV infected cells (Fig. 3b, Additional file 1: Figure S1a, S1b). To confirm that PFV-triggered ER stress is related to PFV-induced autophagy, we used siRNAs to knock down endogenous GRP78 expression that could block ER stress as the infection progressed. As shown in Fig. 3c, blocking ER stress using siGRP78 significantly suppressed the conversion of LC3-I to LC3-II in PFV-infected BHK-21 cells compared to siRNAs of negative control group. This suggested that PFV-induced autophagy could be inhibited by blocking ER stress. In addition, the expression of p62 was increased in the PFV-infected cells treated with 4-phenyl butyric acid (4-PBA, an inhibitor of ER stress) (Fig. 3d). Meanwhile, the expression of Tas was increased in cells pretreated with 4-PBA. In contrast, pre-treatment of cells with dl-dithiothreitol (DTT, an inducer of ER stress) promoted the conversion of LC3-I to LC3-II and the consumption of endogenous p62 remarkably. At the same time, expression of the viral protein Tas was evidently restricted in cells treated with DTT (Fig. 3d). These results showed that PFV infection triggered ER stress, which might be the mechanism of autophagy induction. Furthermore, PFV-induced autophagy though ER stress might regulate PFV replication.

### PFV induces autophagy by activating ER stress-related UPR signaling

**Fig. 4:**
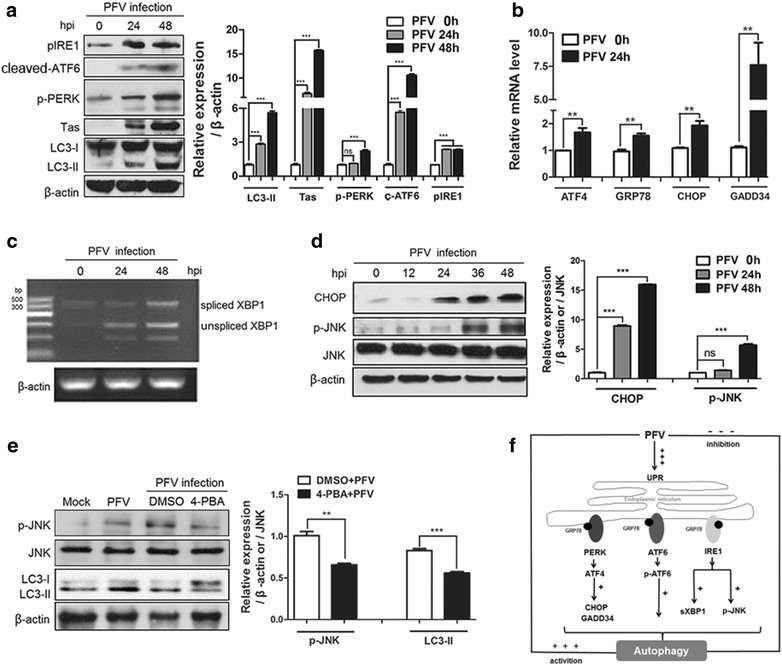
ER stress-related UPR signaling was activated as PFV infection progressed. **a** BHK-21 cells were infected with PFV to analyze ATF6, JNK and PERK phosphorylation and IRE1 activation by western blotting. Cells were infected with PFV at an MOI of 0.5. After PFV infection, cell samples were harvested at 0, 24 and 48 h, and cell extracts were evaluated by western blotting. β-actin was used as the loading control. **b** BHK-21 cells were seeded in 6-well plates and infected with PFV for 24 h (MOI = 0.5). Then, the total RNA (2 μg) was reverse transcribed to cDNA. Q-PCR was used to examine the relative expression (normalized to β-actin) of ER stress sensors such as CHOP, GADD34, ATF4, and GRP78. **c** BHK-21 cells were seeded in 6-well plates and infected with PFV for 0, 24 and 48 h (MOI = 0.5). The mRNA levels of spliced XBP1 and unspliced XBP1 were measured by RT-PCR. DNA agarose gel electrophoresis revealed the mRNA levels of spliced XBP1 and unspliced XBP1 in the presence or absence of PFV infection. **d** Western blotting was used to analyze the expression of CHOP and JNK signaling in PFV-infected cells. At 0, 12, 24, 34, 48 h after infection with PFV (MOI = 0.5), the cells were subjected to western blotting with the indicated antibodies. **e** Western blotting was used to analyze the expression of LC3 and JNK signaling in PFV-infected cells in the absence or presence of the ER-stress inhibitor 4-PBA. BHK-21 cells were pretreated with 4-PBA for 4 h, followed by infection with PFV (MOI = 0.5). Then cells were further cultured in maintenance medium in the absence or presence of 4-PBA. At 48 h after infection with PFV, the cells were subjected to western blotting with the indicated antibodies. As the control groups, BHK-21 cells were infected with mock or PFV (MOI = 0.5). Then cells were further cultured in maintenance medium for 48 h. **f** The graphical illustrated principle signaling pathways involved in PFV-induced autophagy via the ER stress-related UPR and the summary of regulation between PFV replication and PFV-induced autophagy. Significance was analyzed with a two-tailed Student’s *t* test. ^ns^ *P* > 0.05, **P < 0.01, ***P < 0.001

To maintain ER homeostasis, cells have evolved an adaptive response known as the unfolded protein response (UPR) pathway, which refolds or degrades unfolded/misfolded proteins to maintain ER homeostasis and stimulates autophagy [26]. Three ER transmembrane-proximal sensors that act as UPR transducers are protein kinase R-like endoplasmic reticulum kinase (PERK), activating transcription factor 6 (ATF6) and inositol-requiring enzyme-1 (IRE1) [27, 28]. In response to the ER stress, the serine/threonine kinases PERK and IRE1 are activated by auto-phosphorylation, while ATF6 acts as transcription factor and is cleaved to its active isoform by trans-membrane proteases. To determine whether PFV-induced autophagy can be attributed to ER stress-mediated UPR signaling, we measured the activation of three UPR pathways during PFV infection. As shown in Fig. 4a, PERK and IRE1 were phosphorylated and cleaved ATF6 was increased as the process of PFV infection, suggesting that these three pathways were activated to trigger PFV-induced autophagy initiation. To further confirm whether PFV-induced ER stress could be attributed to the PERK pathway, we measured three markers of the PERK pathway: cyclic AMP-dependent transcription factor 4 (ATF4), growth arrest and DNA damage-inducible protein 34 (GADD34) and CCAAT/enhancer binding protein homologous protein (CHOP) [29–31]. PFV infection significantly increased ATF4/GADD34/CHOP transcription after 24 h (Fig. 4b), implying PERK activation. At the same time, the activation of the IRE1 pathway led to the production of X box-binding protein 1 (XBP1) transcription factor via splicing un-spliced XBP1 (uXBP1) mRNA to create spliced XBP1 (sXBP1) mRNA, which in turn promoted the expression of the full-length XBP1. This splicing of the *XBP1* gene is commonly used as readout of UPR activation [32, 33]. We found that the spliced form of XBP1 mRNA was increased as the process of PFV infection, and this suggested that PFV infection activated UPR signaling (Fig. 4c). In addition, IRE1 can also activate the cellular c-Jun N-terminal kinase (JNK) pathway to trigger autophagy in response to ER stress [24, 34]. We found that JNK phosphorylation was increased during PFV infection at 36 and 48 h (Fig. 4d). Conversely, as shown in Fig. 4e, inhibition of ER stress by 4-PBA in PFV-infected cells decelerated the phosphorylation of JNK and the conversion of LC3-II relative to untreated controls (Fig. 4e). This indicated that the activation of downstream sensors such as JNK may participate in PFV-induced autophagy. Altogether, these results indicated that PFV infection activates the PERK, ATF6, and IRE1 pathways of the UPR (Fig. 4f).

Induction of autophagic vesicles formation in infected cells can be driven by recognition of viral RNA by innate immune sensors, virus binding to receptors, expression of viral proteins that usually leads to the induction of UPR due to ER stress and/or production of Reactive Oxygen Species (ROS) [35]. We have confirmed that PFV infection also triggered ER-stress related UPR. It was likely that ER-stress UPR signaling partly contributed to PFV induced complete autophagy. It has been reported that NDV (Newcastle disease virus) can induce autophagy, which is associated with activating the ER-stress-related UPR by viral proteins to benefit its replication [36]. In addition, Datan et al. found that dengue virus could up-regulate ER stress and ataxia telangiectasia mutated (ATM) signaling followed by the production of ROS to enhance autophagy, and the increased autophagy enabled dengue to reproduce [29]. However, our results showed that promoting PFV-induced autophagy though ER stress suppressed the viral replication (Fig. 3d). This implies a novel mechanism for regulating PFV replication caused by PFV-induced autophagy via ER-stress-related UPR signaling.

### PFV-induced autophagy negatively regulates viral replication

**Fig. 5:**
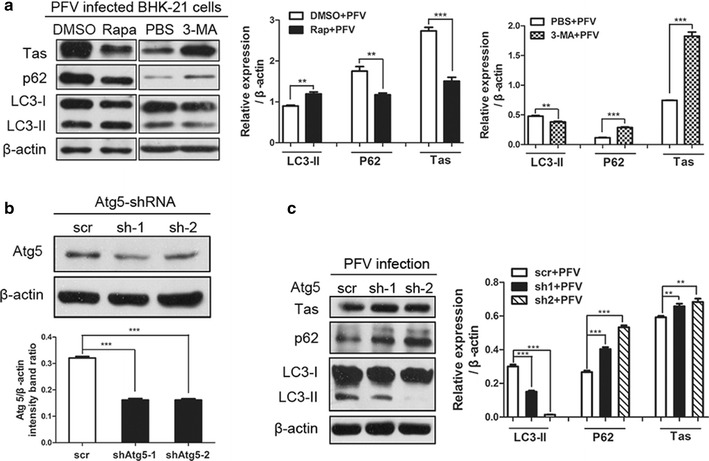
Inhibition of autophagy promotes PFV replication, and activation of autophagy reduces its replication. **a** Western blotting was used to analyze the expression of the viral protein Tas in PFV-infected cells in the absence or presence of 3-MA or Rapa. BHK-21 cells were pre-treated with 3-MA (10 mM) or Rapa (400 nM) for 4 h, followed by infection with PFV at an MOI of 0.5. The intracellular virus yields were determined by measuring viral protein at 24 hpi. **b** Western blotting was used to analyze the expression of Atg5 in cells that down-regulated Atg5 in response to specific shRNAs. BHK-21 cells were transfected with Atg5-specific shRNA1 or shRNA2 for 48 h. The cell samples were harvested and lysed, and cell extracts were measured by immunoblotting with anti-Atg5 antibody. **c** BHK-21 cells were transfected with Atg5-specific shRNA1 or shRNA2 for 48 h and then infected with PFV (MOI = 0.5) for another 24 h to analyze the expression of the viral protein Tas in PFV-infected cells that down-regulated Atg5 in response to specific shRNAs. Significance was analyzed with a two-tailed Student’s *t* test. **P < 0.01, ***P < 0.001

The above results showed that PFV-induced complete autophagy might involving in ER-stress-related UPR signaling. However, we wanted to further clarify the effect of PFV-induced autophagy on viral replication. To investigate this question, BHK-21 cells were pretreated with rapamycin (Rapa, an autophagy inducer) or 3-methyladenine (3-MA, an autophagy inhibitor) and infected with PFV for 24 h. We observed a significant decrease in Tas levels upon Rapa treatment (Figs. 3d, 5a). In contrast, Tas was significantly increased in the cells treated with 3-MA compared to the controls (Fig. 5a). Furthermore, we used shRNAs to silence autophagy-related gene 5 (Atg5), an autophagy essential gene critical for the elongation of the phagophore membranes. ShRNA-mediated Atg5 silencing could inhibit autophagosome formation in the early stage of autophagy [16, 17]. Endogenous levels of Atg5 were knocked down significantly in BHK-21 cells transfected with shRNAs (Fig. 5b). Similar to the results with 3-MA treatment, down-regulation of Atg5 effectively resulted in an increase in the expression of the viral protein Tas compared to the control (Fig. 5c). Therefore, PFV replication was enhanced by inhibition of autophagy and reduced by the activation of autophagy.

Recently, autophagy as a cellular adaptive response has been found to be involved in various viral infections [37]. Inhibition of PFV-induced autophagy could reinforce PFV replication and enhancing this process could attenuate viral replication, suggesting that autophagy negative regulated PFV replication and might play an antiviral role in PFV infection. PFV-induced complete autophagy was a process that the autophagosome fuses with a lysosome to form an autolysosome where the captured cytosol component and the inner membrane were degraded [18].

## Conclusions

In this paper, we report for the first time that PFV infection induces the complete autophagic process to promote autophagosome accumulation. Activation of the ER stress-related UPR contributes to PFV-induced autophagy, and PFV-induced autophagy may act as an antiviral mechanism by negatively regulating PFV replication.

## Methods

### Cells, viruses and plasmids

BHK-21 cells were stored by our laboratory. BHK-21 cells were maintained in minimum essential medium (MEM) supplemented with 10% fetal bovine serum (FBS) (SV30087; HyClone). The cells were cultured at 37 °C in a humidified incubator with 5% CO_2_.

The PFV virions were acquired by transient transfection 293T cells with the pHSRV13 proviral plasmid using the PEI transfection reagent [38, 39]. Mock-infected cells were similarly produced by transient transfection of pAT153 blank vector using the PEI transfection reagent to generate negative controls [39]. The cells and the culture medium were freeze-thawed for three cycles to release viruses at 48 h after transfection. To prepare cell-free virus stocks or mock-infected supernatant, the culture supernatant was centrifuged at 4000×*g* for 10 min and filtered through a 0.22 μm-pore-size filter membrane, and stored at −80 °C. To assess the viral titer, the BHK-21 cells were seeded into 96-well plates, and the medium was removed after the cells were incubated with virus for 1.5 h in an incubator. Then, the supernatant was replaced with growth medium and maintained for 48 h. Virus titers were calculated as 50% tissue culture infectious doses (TCID50) using the Reed-Münch method [40].

The following plasmids were constructed by our laboratory: cDNA encoding LC3 was cloned into pEGFP-N1 (Clontech, #6085-1); fragments encoding PFV-Bet and Tas were cloned into pCMV-HA (Clontech, #631604); cDNAs encoding PFV-Gag were cloned into pCMV-His (Clontech); pLKO.1-TRC (Addgene, #10878). The primers are shown in Additional file 2: Table S1. The tandem fluorescent monomeric red fluorescent protein mRFP-GFP-LC3 (ptfLC3, Addgene, #21074), and GFP-LAMP1 were purchased from Addgene. The infectious pHSRV13 provirus DNA and the empty plasmid pAT153 were a gift from Professor Rolf M. Flügel (German Cancer Research Center) [41]. GRP78-specific siRNA (5′-GGAGCGCAUUGAUACUAGATT-3′) and a non-silencing siRNA (NC) (5′-UUCUCCGGACGUGUCACGUTT-3′, used as a negative control) were purchased from (GenePharma Shanghai, China) [42]. Plasmids and siRNA transfections were performed by using lipofectamine 2000 reagent (Life Technologies, Grand Island, NY, USA) according to the manufacturer’s instructions.

### Reagents and antibodies

Chloroquine (CQ), Rapamycin (Rapa) and 3-methyladenine (3-MA) were purchased from Sigma-Aldrich. Dimethyl sulfoxide (DMSO), dl-dithiothreitol (DTT) and 4-phenyl butyric acid (4-PBA) were purchased from Biosharp. Rabbit anti-LC3, anti-Atg5, anti-p62/SQSTM1, and anti-HA polyclonal antibodies (Abs) were purchased from Cell Signaling Technology. Anti-phospho-JNK/JNK, anti-phospho-ERK1/2pY204, anti-phospho-IRE1, anti-phospho-ATF6 and anti-β-actin polyclonal Abs were purchased from Abcam. Antibody against PFV Gag was kindly provided by Professor Li Zhi, and anti-Tas was produced by immunizing mouse with prokaryotic expressed Tas and purified according to standard procedures [43]. HRP-conjugated goat anti-mouse or HRP-conjugated goat anti-rabbit secondary antibodies were from PM BIOPRIMACY.

### Viral infection and drug treatment

BHK-21 cells were seeded into 6-well or 12-well plates and cultured until 80% confluency was reached. Then, the cells were infected with PFV at a MOI of 0.5. After 1.5 h infection, the cells were washed three times with phosphate-buffered saline (PBS) to remove unattached viruses and were incubated in maintenance medium (2% FBS) at 37 °C for the indicated time. BHK-21 cells were pretreated with optimal concentrations of CQ (50 μM), Rapa (400 nM), DTT (1 mM), or 4-PBA (1 mM) for 4 h, followed by infection with PFV. The cells were further cultured in maintenance medium in the absence or presence of drugs. For 3-MA (10 mM) treatment, cells were pretreated for 2 h with 3-MA, followed by infection with PFV. The cells were further cultured in maintenance medium in the absence or presence of 3-MA until the samples were harvested.

### UV-inactivated PFV

PFV supernatant was prepared as methods above. PFV supernatant was radiated with UV (254 nm) for 1.5 h. BHK-21 cells were incubated with UV-inactivated PFV supernatant for 1.5 h in an incubator. Then, the supernatant was replaced with growth medium and maintained for indicated time. The inactivation efficiency of cells which were infected with UV-inactivated PFV was measured by western blotting with specific viral protein Gag antibody.

### Transmission electron microscopy (TEM)

BHK-21 cells were collected at 24 hpi with PFV at an MOI of 0.5. Cells were fixed with 2.5% glutaraldehyde in 0.1 M phosphate buffer (pH 7.4) overnight and subjected to preparation for TEM observation [25]. Autophagosomes were defined as double- or single-membrane vesicles measuring 0.3–2.0 μm in diameter.

### Confocal fluorescence microscopy

BHK-21 cells were seeded in 12- or 24-well plates that contained coverslips and were grown to 70% confluence. Then, BHK-21 cells were transfected with GFP-LC3, ptfLC3 or GFP-LAMP1 using Turbofect (Thermo Fisher #R0531) according to the manufacturer’s guideline. The cells were infected with PFV or treated with drugs as described above at 24 hpi. Treated cells were washed twice with PBS and fixed in 4% paraformaldehyde in PBS for 15 min. Coverslips were inverted onto slides containing 50% glycerol, and fluorescence signals were visualized with a confocal fluorescence microscope (Leica-LCS-SP8-STED) or fluorescence microscope.

### Western blotting

Following the indicated treatment or transfection, BHK-21 cells were washed twice with ice-cold 1× phate-buffered saline (PBS) and lysed on ice with RIPA buffer (Beyotime Biotechnology #P0013B) containing a 1× protease inhibitor cocktail. Thereafter, the cell lysates were centrifuged at 13,000 rpm for 15 min at 4 °C. The samples were boiled at 100 °C for 10 min in sample loading buffer (5% SDS, 10% glycerol, 60 mM Tris pH 6.8, 5% β-mercaptoethanol, and 0.01% bromophenol blue) before electrophoretic separation. The protein samples were resolved by 12.5 or 10% sodium dodecyl sulfate–polyacrylamide gel electrophoresis (SDS-PAGE) and transferred to polyvinylidene fluoride (PVDF) membranes (Roche). The membranes were blocked in 5% nonfat milk-TBST for 3 h at room temperature. Next, the membranes were incubated with primary antibodies overnight at 4 °C followed by washing with 1× TBST for 10 min × 3 times. Then the membranes were hybridized with horseradish peroxidase (HRP)-conjugated secondary antibody (Tianjin Sungene Biotech Co., Ltd) for 1.5 h at room temperature. Antibody–antigen complexes were observed using enhanced chemiluminescence (ECL) system (Advansta, Menlo Park, CA, USA) with a Kodak imager (Carestream Health). The quantitative analysis of the relative intensities of proteins (normalized to β-actin) was performed with Quantity One Software (Bio-Rad, Hercules, CA, USA) and GraphPad Prism 5. All data are representative of three independent experiments with triplicate samples. Statistical significance was analyzed with a two-tailed Student’s *t* test. *P < 0.05, **P < 0.01, ***P < 0.001. All experiments in this study are repeated at least for three times.

### qPCR

Quantitative PCR (qPCR) was used to determine the relative quantities of RNA (cDNA) and DNA. Total RNA was extracted from harvested cells using Trizol reagent (Invitrogen, Carlsbad, CA, USA), and cDNA was obtained by reverse transcription with the Revert Aid™ First Strand cDNA Synthesis Kit (Thermo Scientific, Rockford, USA) according to the manufacturer’s protocol. QPCR was performed with a SYBR green Real-Time PCR master mix kit (Toyobo) according to the manufacturer’s protocol. All primers are listed in Additional file 2. The program set on the CFX96 sequence detection system (BIO-RAD) was 95 °C for 30 s, followed by 40 cycles at 95 °C for 15 s, 58 °C for 20 s, and 72 °C for 15 s. Values for the relative quantification were calculated by the ∆∆C_t_ method. Melting curve analysis was performed to verify the specificity of the products, and each sample was tested in triplicate.

### Quantitative measurement of spliced XBP1 mRNA

1 × 10^6^ BHK-21 cells were seeded in 6-well. 48 h after PFV infection, cells were washed three times with phosphate-buffered saline (PBS), and total RNAs were extracted by Trizol reagment (Invitrogen, Carlsbad, CA, USA). Then, 1 μg of total RNA samples were reverse transcribed using the Revert Aid™ First Strand cDNA Synthesis Kit (Thermo Scientific, Rockford, USA) according to the manufacturer’s protocol. For measurement of spliced XBP1 mRNA, XBP1 double-stranded cDNA was synthesized under the following thermal cycling conditions: 94 °C 5 min, 95 °C 30 s–55 °C 30 s–72 °C 30 s 30 cycles. Then, 7.5 U of restriction enzyme *Pst I* (TaKaRa Bio, Shiga, Japan) was added to the reaction mixture for 1 h to digest the double-stranded DNA of unspliced XBP1. The spliced XBP1 DNA wouldn’t be digested. β-actin mRNA expression was used as an internal control. PCR products were analyzed by electrophoresis through 1.5% agarose gel, and their identity was checked by DNA sequencing.

### Statistical analysis

Data were expressed as the means ± standard deviations. Statistical analyses were performed using GraphPad Prism (GraphPad Software, La Jolla, CA, USA) to evaluate the differences between experimental groups. Statistical significance was analyzed with a two-tailed Student’s *t* test. ^ns^ *P* > 0.05, *P < 0.05, **P < 0.01, ***P < 0.001. All data are representative of three independent experiments with triplicate samples. All experiments in this study are repeated at least for three times.

## Additional file

**Additional file 1: Figure S1.** A productive PFV infection is required to induce autophagy. **a** BHK-21 cells were infected with mock supernatant to analyze GRP78, p62 and LC3 protein expression by western blotting. After 1.5 h of mock supernatant incubation at 37 °C, the cells were further cultured in maintenance medium. Then cell samples were harvested at 0, 12, 24, 48 and 72 hpi, and cell extracts were blotted with anti-GRP78, anti-P62, and anti-LC3 antibodies. **b** Before infection, PFV (MOI = 0.5) were radiated with UV for 1.5 h. Then, BHK-21 cells were inoculated with UV-inactivated PFV for 1.5 h at 37 °C. The cells were further cultured in maintenance medium. After UV-inactivated PFV infection, cell samples were harvested at 0, 12, 24, 48 and 72 hpi, and the cell samples were processed and blotted with anti-GRP78, anti-P62, and anti-LC3 antibodies. Quantitation of protein levels from the western blot by using Quantity one software (Bio-Rad); all data are representative of three independent experiments with triplicate samples. Significance was analyzed with a two-tailed Student’s *t* test. ^*ns*^ *P* > 0.05. **c** GFP-LC3 dots were visualized via confocal microscopy. BHK-21 cells were transfected with GFP-LC3 plasmids for 24 h, followed by mock or UV-PFV infection for 24 h, and the GFP-LC3 aggregates in the cells were assessed via confocal microscopy. Scale bars, 10 μm. **d** BHK-21 cells were seeded in 6-well plates and infected with mock supernatant or UV-inactivated PFV for 24 h and 48 h. Then, the total RNA (2 μg) was reverse transcribed to cDNA. Q-PCR was used to examine the relative expression (normalized to β-actin) of ER stress sensors GRP78. **e** BHK-21 cells were pretreated with optimal concentrations of CQ (50 μM), Rapa (400 nM), DTT (1 mM), or 4-PBA (1 mM) for 4 h, followed by infection with mock supernatant. For 3-MA (10 mM) treatment, cells were pretreated for 2 h with 3-MA, followed by infection with PFV or mock supernatant. After 1.5 h of virus absorption at 37 °C, the cells were further cultured in maintenance medium. The cells were further cultured in maintenance medium in the presence of these drugs until the samples were harvested. PFV-infected sample and Mock + DMSO groups were also been analyzed by western blotting. Quantitation of protein levels from the western blot by using Quantity one software (Bio-Rad); all data are representative of three independent experiments with triplicate samples. Significance was analyzed with a two-tailed Student’s *t* test. ^*ns*^ *P* > 0.05, ****P* < 0.001.

**Additional file 2.** Primers used for the construction of various plasmids and qPCR. 

## Abbreviations

ATF4: cyclic AMP-dependent transcription factor 4
ATF6: activating transcription factor 6
ATG: autophagy-related gene
BHK-21: baby hamster kidney cell line
CHOP: CCAAT/enhancer binding protein homologous protein
CQ: chloroquine
DMSO: dimethyl sulfoxide
DTT: dl-dithiothreitol
ER: endoplasmic reticulum
FBS: fetal bovine serum
4-PBA: 4-phenyl butyric acid
GADD34: growth arrest and DNA damage-inducible protein 34
GFP: green fluorescent protein
GRP78/Bip: glucose-regulated protein 78
HIV: human immunodeficiency virus
HPI: hours post-infection
HTLV: human T cell leukemia virus
JNK: Jun N-terminal kinase
IRE1: inositol-requiring enzyme 1
LC3: microtubule-associated protein 1 light chain 3
LAMP1: lysosomal-associated membrane protein 1
MEM: minimum essential medium
MOI: multiplicity of infection
P62: sequestosome-1/ubiquitin-binding protein
PBS: phosphate-buffered saline
PERK: protein kinase R-like endoplasmic reticulum kinase
PFV: prototype foamy virus
Rapa: rapamycine
RFP: red fluorescent protein
ROS: reactive oxygen species
3-MA: 3-methyladenine
UPR: unfolded protein response
XBP1: X box-binding protein 1
