# Supplementary figures and images for: Prototype foamy virus elicits complete autophagy involving the ER stress-related UPR pathway
Source: Retrovirology. 2017 Mar 7;14:16. doi: 10.1186/s12977-017-0341-x (PMC5341167; doi:10.1186/s12977-017-0341-x)

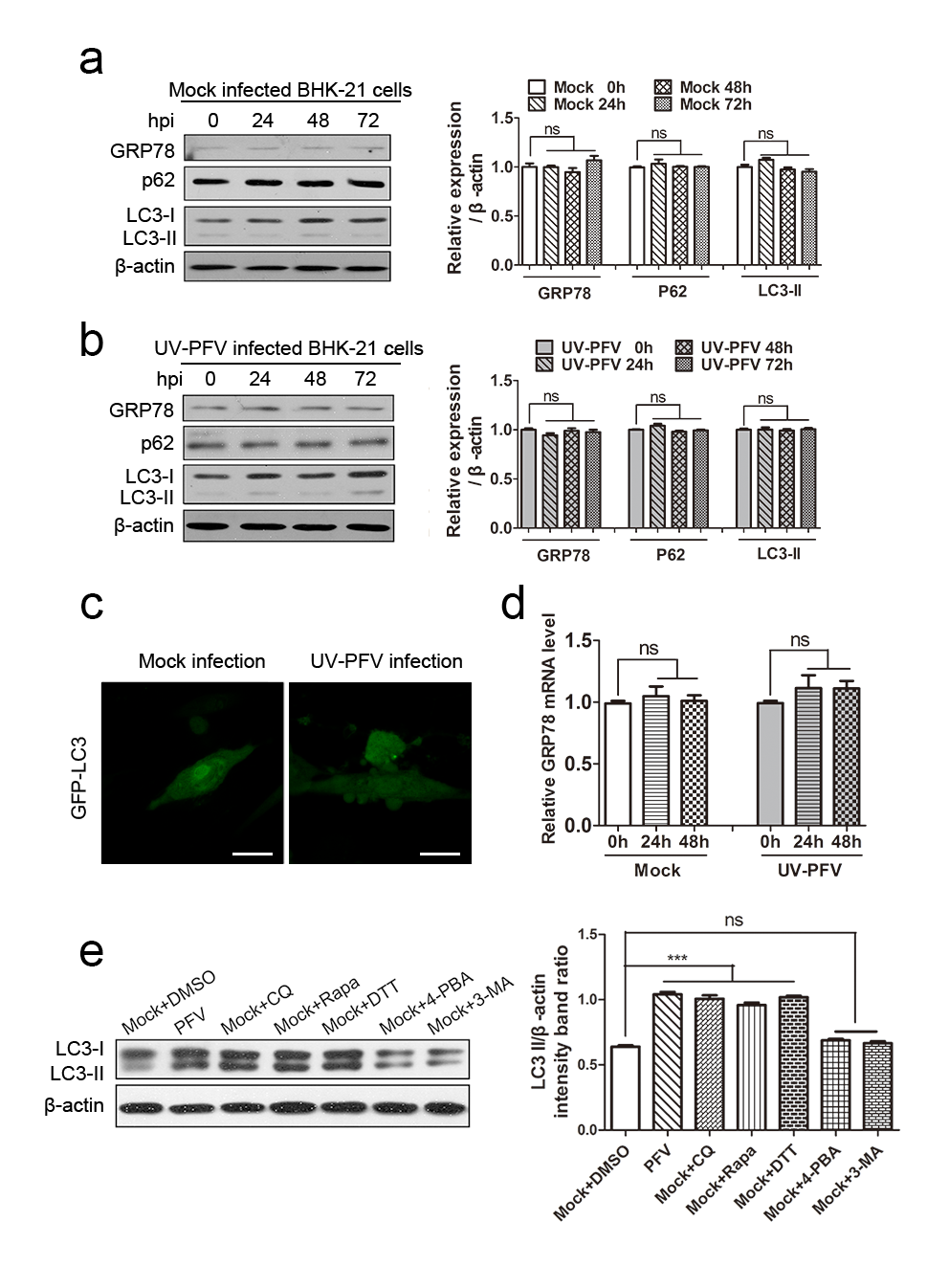

Supplement: Supplementary file 1 — Additional file 1: Figure S1. A productive PFV infection is required to induce autophagy. a BHK-21 cells were infected with mock supernatant to analyze GRP78, p62 and LC3 protein expression by western blotting. After 1.5 h of mock supernatant incubation at 37 °C, the cells were further cultured in maintenance medium. Then cell samples were harvested at 0, 12, 24, 48 and 72 hpi, and cell extracts were blotted with anti-GRP78, anti-P62, and anti-LC3 antibodies. b Before infection, PFV (MOI = 0.5) were radiated with UV for 1.5 h. Then, BHK-21 cells were inoculated with UV-inactivated PFV for 1.5 h at 37 °C. The cells were further cultured in maintenance medium. After UV-inactivated PFV infection, cell samples were harvested at 0, 12, 24, 48 and 72 hpi, and the cell samples were processed and blotted with anti-GRP78, anti-P62, and anti-LC3 antibodies. Quantitation of protein levels from the western blot by using Quantity one software (Bio-Rad); all data are representative of three independent experiments with triplicate samples. Significance was analyzed with a two-tailed Student’s t test. ns P > 0.05. c GFP-LC3 dots were visualized via confocal microscopy. BHK-21 cells were transfected with GFP-LC3 plasmids for 24 h, followed by mock or UV-PFV infection for 24 h, and the GFP-LC3 aggregates in the cells were assessed via confocal microscopy. Scale bars, 10 μm. d BHK-21 cells were seeded in 6-well plates and infected with mock supernatant or UV-inactivated PFV for 24 h and 48 h. Then, the total RNA (2 μg) was reverse transcribed to cDNA. Q-PCR was used to examine the relative expression (normalized to β-actin) of ER stress sensors GRP78. e BHK-21 cells were pretreated with optimal concentrations of CQ (50 μM), Rapa (400 nM), DTT (1 mM), or 4-PBA (1 mM) for 4 h, followed by infection with mock supernatant. For 3-MA (10 mM) treatment, cells were pretreated for 2 h with 3-MA, followed by infection with PFV or mock supernatant. After 1.5 h of virus absorption at 37 °C, the c [file 12977_2017_341_MOESM1_ESM.tif]
