# Supplementary material for: Prototype foamy virus elicits complete autophagy involving the ER stress-related UPR pathway
Source: Retrovirology. 2017 Mar 7;14:16. doi: 10.1186/s12977-017-0341-x (PMC5341167; doi:10.1186/s12977-017-0341-x)
Supplement: Supplementary file 2 — Additional file 2. Primers used for the construction of various plasmids and qPCR. [file 12977_2017_341_MOESM2_ESM.docx]

**Additional file 2:**

Primers used for the construction of various plasmids and qPCR.

| **Primer name** | **Sequence(5’-3’)** | **purpose** |
| --- | --- | --- |
| F-siGRP78 | 5’-GGAGCGCAUUGAUACUAGATT-3’ | for siRNAs of GRP78 |
| R-siGRP78 | 5’-UCUAGUAUCAAUGCGCUCCTT-3’ |  |
| F-siNC | 5’-UUCUCCGGACGUGUCACGUTT-3’ | for siRNAs of GRP78 Negative control |
| R-siNC | 5’-ACGUGACACGUUCGGAGAATT-3’ |  |
| F-XBP1 | 5’-GCGCTGAGGAGGAAACTG-3’ | for XBP1 RT-PCR |
| R-XBP1 | 5’-GGGAGGCTGGTAAGGAACT-3’ |  |
| F-ATF4 | 5’-GGCCAAGCACTTCAAACCTC-3’ | for ATF4 qPCR |
| R-ATF4 | 5’-GAGAAGGCATCCTCCTTGCT-3’ |  |
| F-GRP78 | 5’-GAACGTCTGATTGGCGATGC-3’ | for GRP78 qPCR |
| R-GRP78 | 5’-TCAACCACCTTGAACGGCAA-3’ |  |
| F-GADD34 | 5’-AAGCTCACAGAACCTCTAC-3’ | for GADD34 qPCR |
| R-GADD34 | 5’-GATGTCCACAGAAGAACTTC-3’ |  |
| F-CHOP | 5’-CGGAAACAGAGTGGTCAGT-3’ | for CHOP qPCR |
| R-CHOP | 5’-TACACTTCCGGAGAGACAG-3’ |  |
| F-actin | 5’-CACGATGGAGGGGCCGGACTCATC-3’ | for β-actin qPCR |
| R-actin | 5’-TAAAGACCTCTATGCCAACACAGT-3’ |  |
|  |  |  |
